# E-TUBE: dielectric waveguide cable for high-speed communication

**DOI:** 10.1038/s41598-020-75363-4

**Published:** 2020-10-26

**Authors:** Ha Il Song, Joon-Yeong Lee, Hyosup Won, Chang-Ahn Kim, Huxian Jin, Jake Eu, Jinho Park, Hyeon-Min Bae

**Affiliations:** 1 Department of Electrical Engineering, Korea Advanced Institute of Science and Technology (KAIST), Daejeon, 34141 Republic of Korea; 2 Point2 Technology Inc, Daejeon, 34051 Republic of Korea

**Keywords:** Electrical and electronic engineering, Electronic devices

## Abstract

The demand for advanced interconnects to satisfy market requirements on bandwidth, cost, and power is ever increasing with the expansion of data centers. An interconnect called E-TUBE is presented as a cost-and-power-efficient all-electrical-domain wideband waveguide solution for high-speed high-volume short-reach communication links. The E-TUBE achieves an unprecedented level of throughput-distance product, bending radius, and channel density without requiring complex manufacturing process. The E-TUBE link demonstrates nearly 25 GHz bandwidth at a carrier frequency of 70 GHz and exhibits a frequency-independent insertion loss of 5 dB/m with a frequency-independent group delay of 4 ns/m. Such loss and delay characteristics independent of frequency enabled broadband data transmission over extended reach compared to conventional waveguide links. The E-TUBE link transmits 25 Gbps NRZ data over 3 m distance using a 70 GHz RF CMOS transceiver IC, which is the state-of-the-art throughput-reach product. This new interconnect is expected to overcome the limitations of existing electrical and optical interconnects and to replace them in high throughput links, including but not limited to, 100/400 Gbps board-to-board communications.

## Introduction

Network traffic in data centers has increased the need for greater communication bandwidth^1–3^ due to Internet of Things (IoT) and video streaming services. In order to cope with ever increasing demand, the advancement of semiconductor technology exemplified by the Moor’s law should continue its pace shown in the past decades to support higher speed input/output (I/O) interfaces^4–6^. However, conventional mainstream interconnects using conducting material, i.e., copper are displaying their critical bandwidth limitation caused by skin effect^7,8^. Hence, optical interconnects which used to offer high capacity over longer distance is regarded as a promising replacement for the bandwidth scaling. Nevertheless, deploying such optical interconnects to short-reach high-volume links requires significant capital expenses^9,10^. Therefore, the demand for a new type of high-speed interconnect that can overcome aforementioned economical and technical challenges is unprecedentedly high. In this paper, a low-cost low-loss broadband dielectric waveguide solution referred to as an E-TUBE is proposed as an advanced alternative to existing electrical and optical interconnects in high-speed short-reach communication links.

The E-TUBE is a metal film laminated rectangular dielectric waveguide. E-TUBE link achieves nearly 25 GHz bandwidth using a carrier frequency of 70 GHz. Its insertion loss of 5 dB/m and group delay of 4 ns/m are independent of frequency. The plastic waveguides in the past allow limited bending performance since the dielectric cladding cannot prevent the electromagnetic leakage if the bending radius is comparable to the wavelength^11,12^. For that reason, a fully-enclosed metal waveguide is proposed for low bending loss even on the tightly bent condition^13,14^. However, the boundary condition determined by the fully-enclosed metal cladding causes a nonlinear phase response that results in group velocity dispersion (GVD), which eventually limits throughput-reach product. The E-TUBE implemented with low-loss dielectric core laminated with low-roughness, high-conductivity metal films overcomes the functional limitations of conventional waveguides and demonstrates low-loss, low-GVD characteristics even in the strictly bended condition. A prototype 3 m E-tube link using a 70 GHz carrier frequency achieves a BER of 10^–12^ with a 25 Gbps PRBS15 data pattern.

## Results

**Figure 1:**
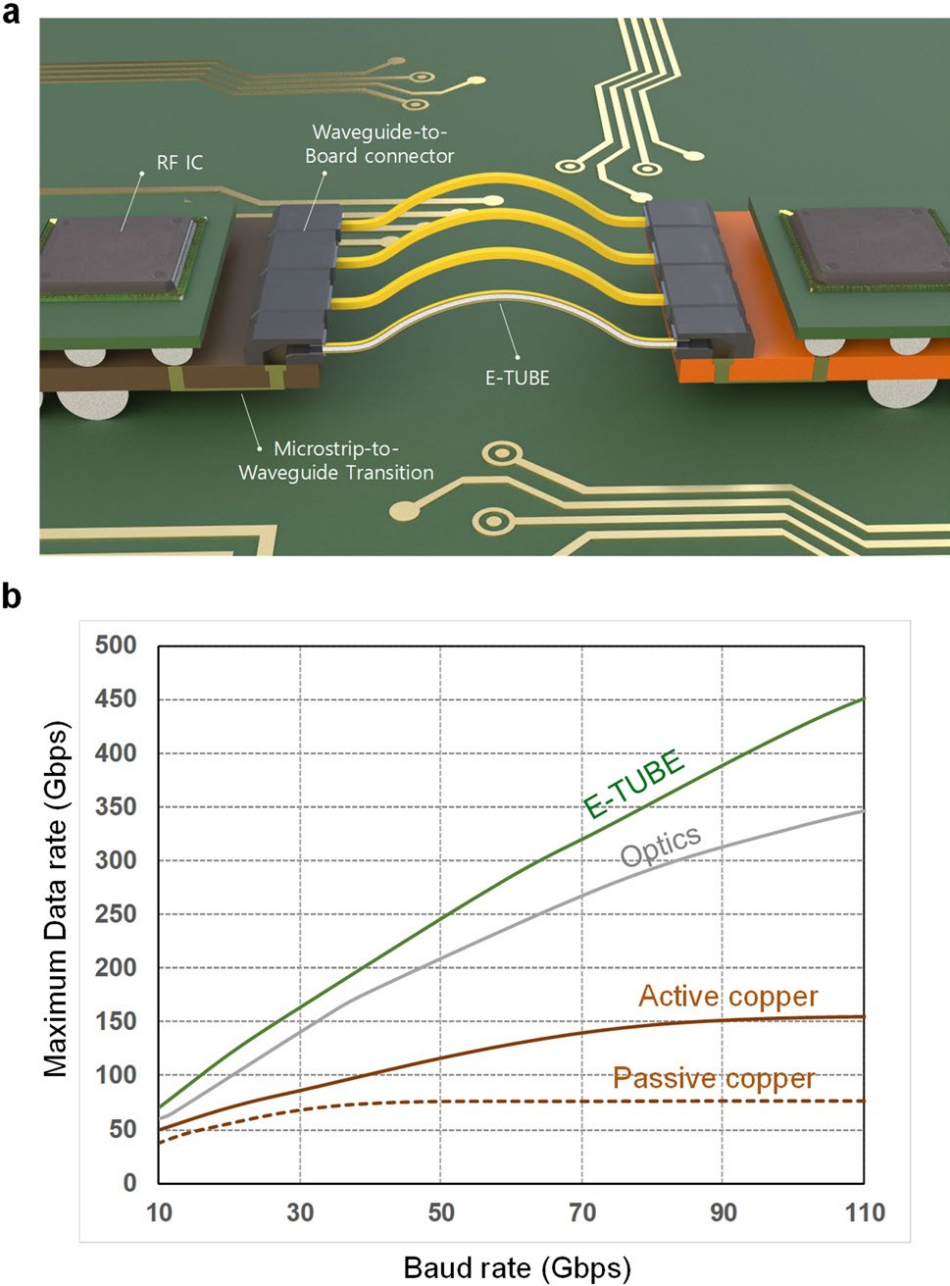
Illustration of E-TUBE links and comparison with electrical and optical links. **(a)** E-TUBE links including metal-clad dielectric waveguides referred to as E-TUBE, the Microstrip-to-Waveguide Transitions on the board, the waveguide-to-board connectors, and RF transmitter/receiver ICs for board-to-board multi-channel communication and **(b)** comparison of maximum transferable data rate of copper, optical, and E-TUBE links. The comparison is based on the general usage of each link.

The overall E-TUBE system is shown in Fig. 1. A CMOS RF transmitter IC up-converts the 25 Gb/s baseband signal into a V-band RF signal. The RF signal is then launched to the waveguide using an on-board Microstrip-to-Waveguide Transition (MWT). The metal-clad dielectric waveguide, E-TUBE, connects the transmitter and receiver boards. The waveguide-to-board connector is implemented using a 90-degree hollow waveguide array arrangement, with each dielectric waveguide inserted horizontally into the corresponding opening slot as shown in Fig. 1. The connector is designed to couple the signal vertically to the board, which increases the area efficiency of the E-TUBE link defined as throughput/footprint. Such 90-degree redirection achieves low-profile board-edge connection of the E-TUBE. On the receiver side, the E-TUBE signal is down-converted by an RF receiver IC to retrieve the baseband signal.

The maximum practical transferable data rate of the E-TUBE and conventional links are compared^15,16^ (Supplementary Note 1). The loss of E-TUBE is independent of the frequency over the entire frequency range above the cutoff frequency owing to the intrinsic characteristics of the dielectric waveguide. Because the bandwidth of the waveguide is unbounded assuming the modal dispersion is negligible, the bandwidth of the E-TUBE channel is higher than 40 GHz for the case of 3 m-channel length. The dominating source of noise in practical communication links is the active circuits in receiving ICs, including baseband and RF components. In addition, considering the fact that prevalent modulation format of high speed links is Pulse Amplitude Modulation (PAM)^17^, the channel capacity of various links can be estimated with Shannon’s law. Figure 1 shows the maximum data rate of various links with respect to the baud rate. The maximum data rate of the copper link saturates as the baud rate increases since no information can be delivered at high frequencies due to excessive signal loss. The maximum data rate of the optical and the E-TUBE links increases in proportions to the baud rate because band limitation does not exist. Consequently, the E-TUBE can be one of the most promising solutions to support the fast-growing demand on the bandwidth of the high-speed short-reach communication links without excessive capital expenditure unlike optical solutions.

The E-TUBE consists of a 3.5-mm wide and 0.6-mm thick dielectric core laminated with thin metal cladding. It allows the electric field of the propagating mode along the E-TUBE to be polarized vertically in the direction of height. And the MWT should be designed to provide identical direction of the electric field in order to minimize the coupling loss.

**Figure 2:**
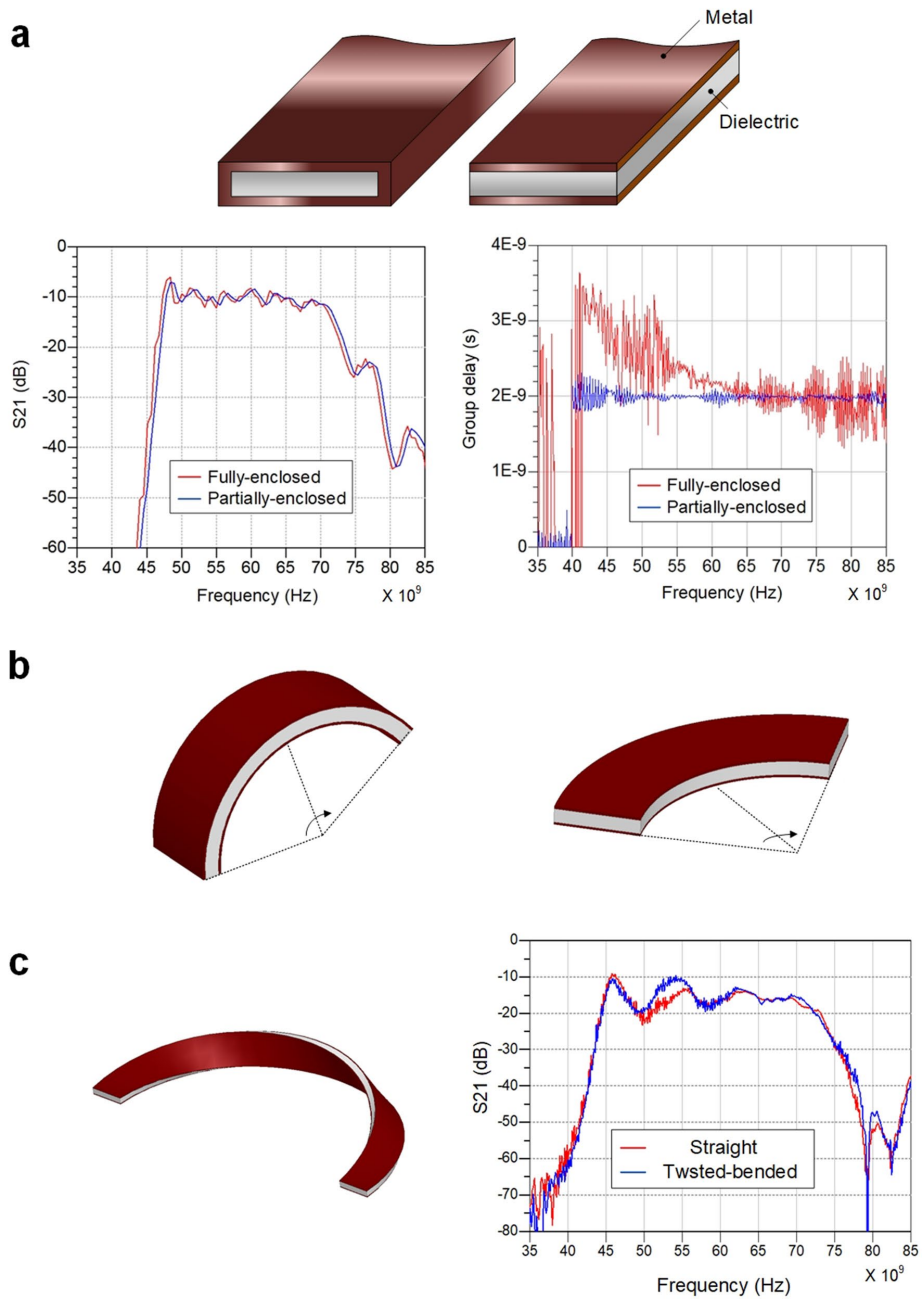
The proposed waveguide and the bending characteristics. (**a**) The structure of the fully-enclosed waveguide and the partially-enclosed waveguide. Comparison of the S21 characteristics and the group delay for the 0.5 m fully-enclosed waveguide and the 0.5 m partially-enclosed waveguide. (**b**) The illustration of the two different bends: E-bend (top) H-bend (bottom). (**c**) The bended waveguide in the H-plane while twisted 180 degree (left). Comparison of S21 characteristics for the straight waveguide and the twisted-bended waveguide (right).

A conventional metallic waveguide that is fully enclosed by the conductor perfectly confines the transverse wave along the waveguide. However, the boundary conditions at the enclosing metal cladding cause frequency-dependent group delay which creates severe channel dispersion^13^. The E-TUBE, in contrast, has thin metal layers laminated only at the top and bottom of the dielectric as shown in Fig. 2. These new boundary conditions lead to a relatively constant group delay over the passband (Supplementary Note 2), which is a significant improvement over conventional designs enabling higher-speed and extended-reach signal transmission. In terms of bending radius, two bending scenarios should be considered since the E-TUBE has a rectangular shape. The bent E-TUBE along the long edges (E-bend) shows negligible performance degradation up to a bending radius of 5 mm because the metal cladding on top and bottom well confines the electromagnetic wave along the E-TUBE. When the E-TUBE is bent along the short edges (H-bend), 3D electromagnetic simulation results show that the minimum bending radius is 20 mm. The refracted wave at the air-dielectric boundary escapes the E-TUBE when the bending radius is less than 20 mm; hence, the bending loss gradually increases. In addition, sharp H-bend below 20 mm can be accompanied by significant physical deformation or even structural damage on the E-TUBE due to the high aspect ratio (3.6:0.5). The bending performance of the E-TUBE can be improved by twisting the E-TUBE 180°, which yields negligible directional dependency without structural deformation under sharp bending.

**Figure 3:**
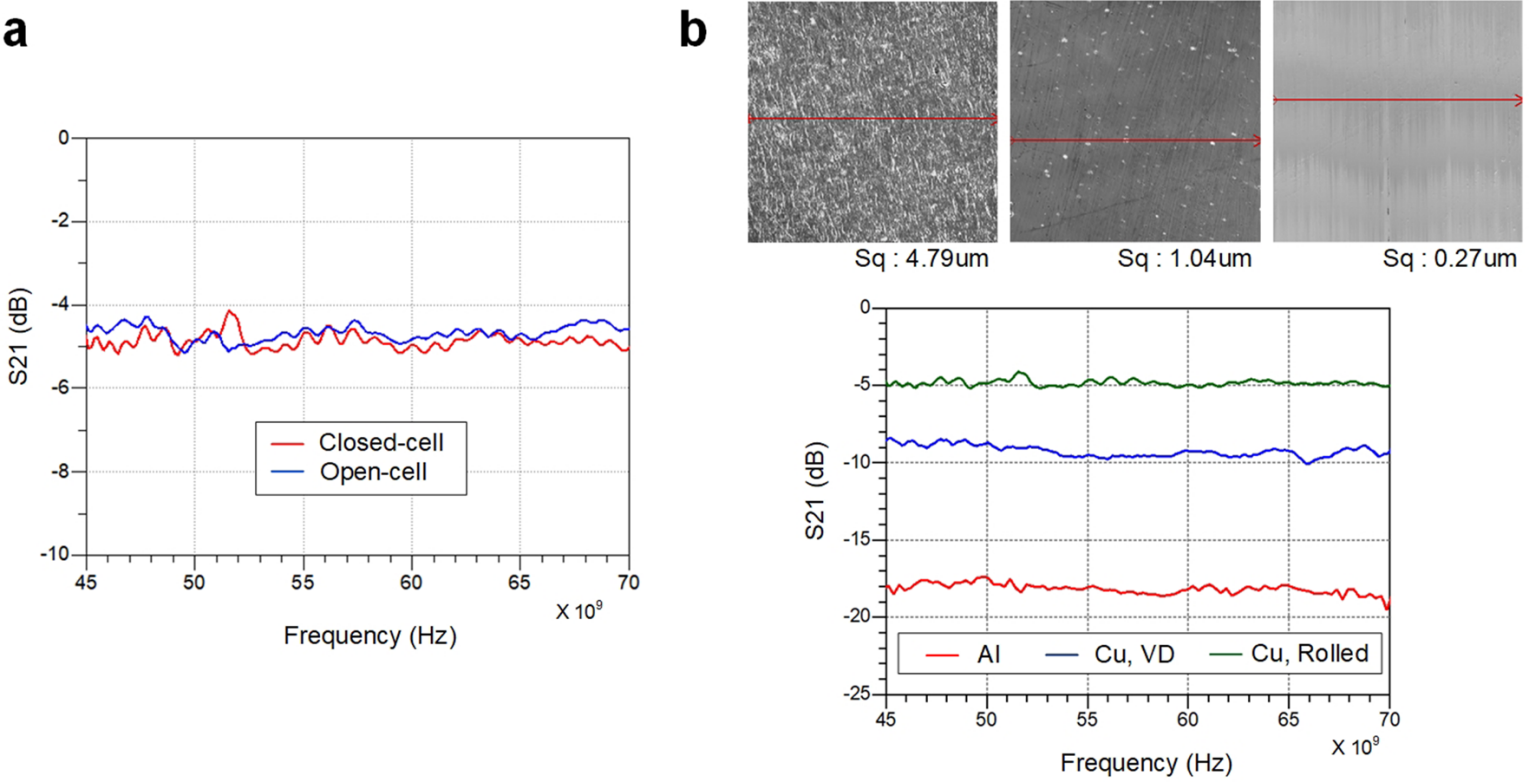
Low-loss metal-clad dielectric waveguide. (**a**) The photograph of the open cell foam and the closed cell foam. Comparison of S21 characteristics for the open cell and the closed cell foam. The loss profile of each waveguide can be achieved by utilizing the two different length of the waveguide channel from the transition at the transmitter side to the one at the receiver side. (**b**) The photograph of the surface of the aluminum film (left), the vapor deposition copper film (middle), and the rolled copper film (right). Comparison of S21 characteristics for the three types of metal film showing the effects of conductivity and surface roughness.

The material of the dielectric core and the metal cladding must be chosen to minimize the propagation loss of E-TUBE while considering its flexibility and manufacturability. Firstly, a low-loss high-elastic dielectric material is required to satisfy the electrical and physical characteristics. A PTFE is commonly known for having a low loss index ($tan\delta =1{e}^{-4} @1GHz$) among polymers^18,19^. However, with increasing frequency, the loss index rapidly increases such that the dielectric loss at 70 GHz is higher than 10 dB/m, which makes the PTFE inappropriate for transmission over a few meters^18^. In order to reduce the loss at high frequencies, foaming processes can be used to form air bubbles within the dielectric. Foams are classified into two groups: open cell foams and closed cell foams. The closed cell foams consist of enclosed, tiny and closely packed air pockets leading to the overall foams more flexible and resilient compared to the open cell foams. These characteristics enable the dielectric core to achieve high dimensional stability after compression, bending, or twisting during the manufacturing process. Figure 3 shows the loss comparison between the open cell PTFE foams and the closed cell PTFE foams. When the effective foaming degrees of two different foams are identical, they demonstrate identical levels of loss. This is because the dimensions of the air bubbles in both foams are several orders of magnitude lower than the wavelength of the signal and thus the difference in the scattering loss is negligible. Secondly, a high-conductivity and low-roughness metal film is required to minimize the conductor loss of the E-TUBE. The electromagnetic field of the propagating wave induces a current on the surface of the metal cladding. The induced electrical energy is dissipated in the form of heat due to the resistivity of the metal cladding that is affected by conductivity and surface roughness. As such, a high-conductivity low-roughness metal film should be utilized to minimize the ohmic and scattering losses of the metal surfaces. Figure 3 shows the loss profile of the Cu-clad waveguide and the Al-clad waveguide, wherein we can clearly compare the conductor loss of two different metal films, copper ($\sigma =5.8{ \times 10}^{7}$) and aluminum ($\upsigma =3.8{ \times 10}^{7}$). Given that the surface profiles of the two metals are perfectly identical, the simulated results show that the difference of the conductor loss of the two waveguides is 1.5 dB/m. However, the measured results show that the conductor loss of the Cu-clad waveguide is 9 dB/m lower than that of the Al-clad waveguide, which is caused by the difference in the surface roughness of the copper film (${S}_{q}=0.27 um$) and the aluminum film (${S}_{q}=4.79 um$), where ${S}_{q}$ is the root mean square of the roughness over the measured area. As frequency increases, the skin depth of the metal is gradually reduced and becomes comparable to the surface roughness of the metal at millimeter-wave frequency ranges^20^. As a result, the scattering loss caused by surface roughness is considerably high even for a transmission over a few meters. Figure 3 shows the loss profile of the waveguides with the rolled Cu film (${S}_{q}=0.27 um$) and the vapor deposition Cu film (${S}_{q}=1.04 um$). Typically, the vapor-deposited Cu film shows lower thickness and relatively uniform surface profile compared to the rolled copper film. However, the microporosity defects can be generated due to nodules and the non-uniform growth over the large area with increasing deposition, which eventually enlarges the scattering loss and the electromagnetic leakage. Consequently, the vapor deposited Cu-clad waveguide shows higher conductor loss compared to the rolled Cu-clad waveguide. On the other hand, the low-roughness metal film hardly adhere to the dielectric core, causing delamination. A high-peel strength adhesive is therefore required to maintain the adhesion of the metal film and the dielectric core in preparation of repeated bending.

**Figure 4:**
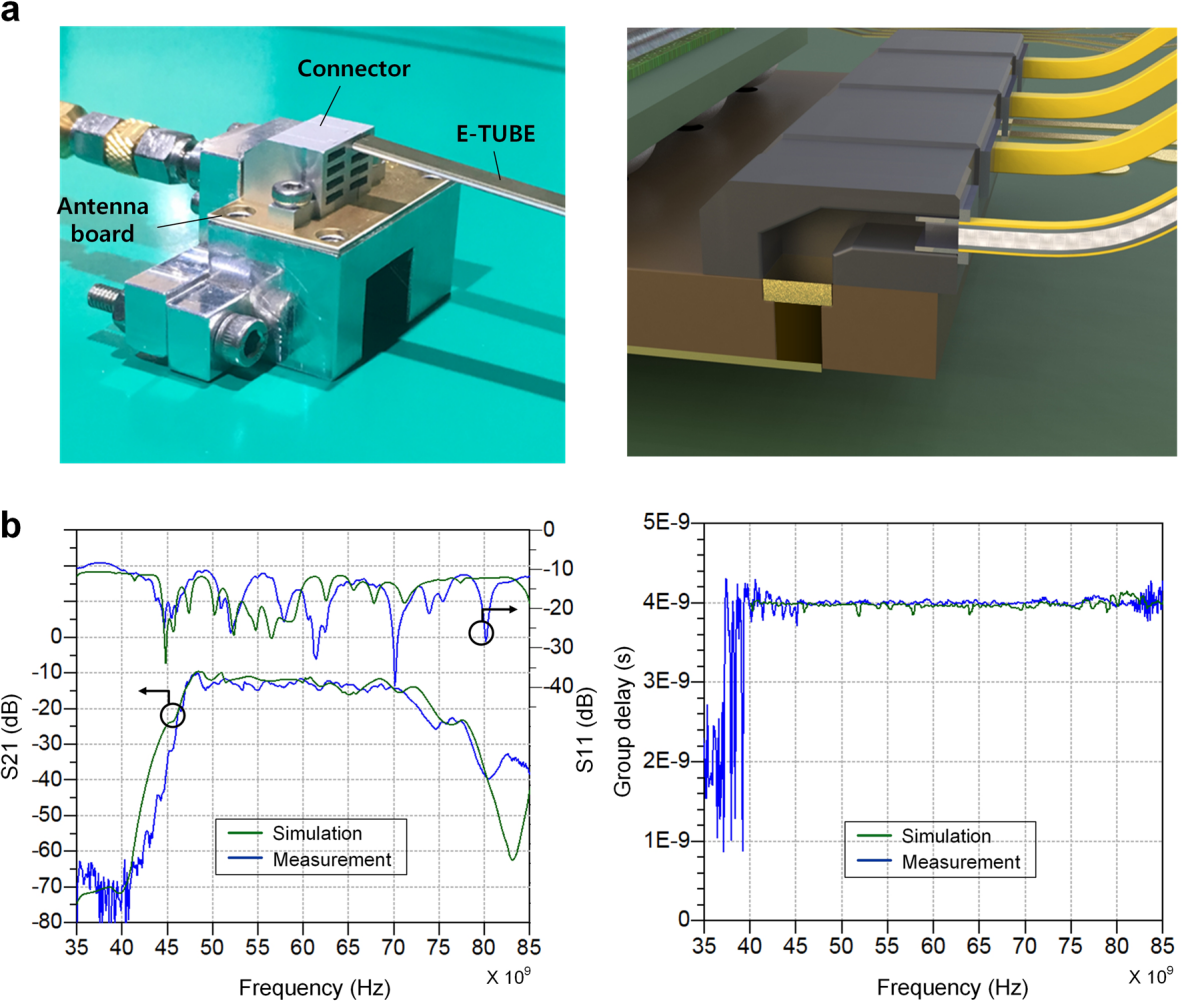
Board-to-E-TUBE interface and E-TUBE channel characteristics. (**a**) The photograph of the board-to-E-TUBE interface (left). The antenna board is mounted on the jig and the board-to-waveguide connector is utilized to couple the radiated signal from the antenna board to the E-TUBE and vice versa. The bolt connection is adopted to mount the board and the connector on the jig. The illustration (right) shows the cross section of the board-to-ETUBE interface. (**b**) Measured S-parameter (left) of a 1.0 m E-TUBE channel including MWTs at both end sides. Measured group delay (right) of the same E-TUBE channel.

Figure 4 shows the interface between the board and the E-TUBE, including the MWT, the board-to-waveguide connector, and the E-TUBE. The slot-coupled MWT is designed to achieve wideband characteristics while suppressing the reflection occurring at the discontinuities between the microstrip line and the waveguide^13^. The E-TUBE is plugged into the opening of the connector and pushed all the way to the MWT on the board, which minimizes the reflection at the boundary between the board and the E-TUBE. The connector is made of the aluminum and the dimensions of the hollow waveguides are the same as those of the E-TUBE, which allows for high confinement and low reflection at the boundary. The frequency response of the E-TUBE channel is shown on the Fig. 4. The return loss is under − 10 dB in the frequency range of 40 GHz to 85 GHz. The measured insertion loss is 13 dB at 70 GHz for a 1 m E-TUBE channel and increases in proportional to the distance at a rate of 5 dB/m. The E-TUBE exhibits frequency-independent group delay of 4 ns/m, which is enabled by the boundary condition of the partially enclosed metal waveguide.

**Figure 5:**
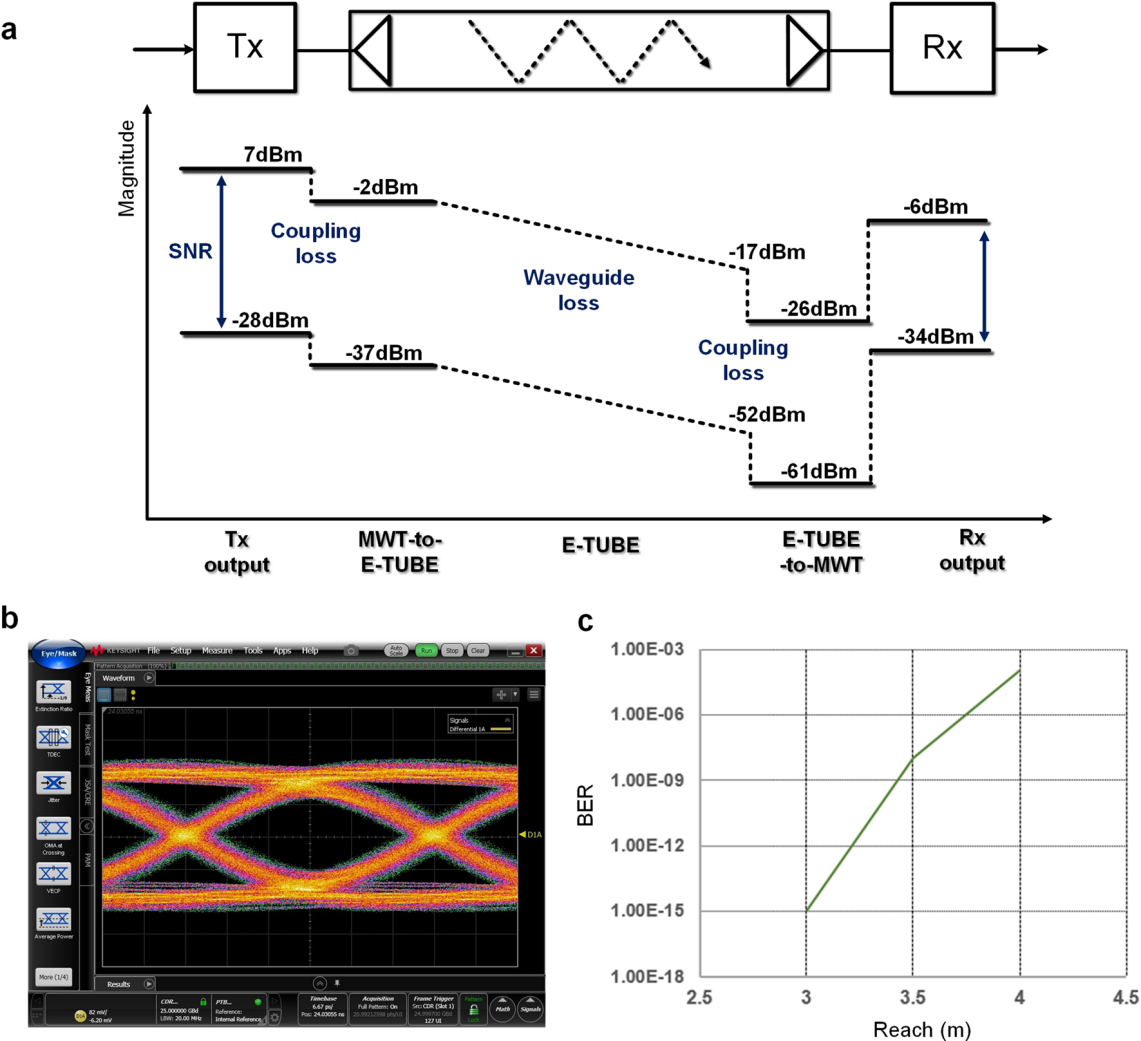
Link budget analysis and eye diagrams, bit-error-rate measurement. (**a**) Link budget of 3 m-E-TUBE interface. The magnitude of signal and noise is presented from the transmitter output to the receiver output. (**b**) The eye diagrams of the receiver output at 25Gbps with PRBS length of 2^15^–1. (**c**) Bit-error-rate measurements with the PRBS patterns of 2^15^–1.

To demonstrate the data transmission through the E-TUBE channel, the RF transceiver IC fabricated in 28 nm CMOS up and down converts the baseband signal with a 70 GHz carrier to utilize the passband of the E-TUBE channel (Supplementary Note 3). The RF transmitter consists of an up-conversion mixer and a power-amplifier while the RF receiver consists of a low-noise amplifier and a down-conversion mixer. Two 70 GHz sinusoidal signals are internally generated at the transmitter and the receiver IC respectively. Due to a sharp cutoff response at the upper corner frequency, only the lower sideband of the modulated signal is transmitted, which enables 2x-bandwidth-efficient data transmission as compared to the conventional wireless communication schemes^12^. The link budget analysis shown on Fig. 5 indicates the signal-to-noise ratio (SNR) along the 3 m E-TUBE interface. The receiver output SNR is 28 dB, which guarantees the error-free operation with 25 Gbps NRZ transmission. Figure 5 shows the eye diagram of the receiver output at 25 Gbps with a pseudorandom binary sequence (PRBS) pattern of 2^15^–1 conducted with a 3 m E-TUBE channel. The measured BER is less than 10^–12^ for the channel reach less than 3 m.

## Discussion

The E-TUBE link demonstrates the unprecedented level of performance in terms of throughput-distance product, bending performance, and channel density. The loss reduction due to the low-loss dielectric and low-profile copper film enables a high SNR of the receiver up to 3 m, a significant improvement over existing plastic waveguide solutions. The partially enclosed metal structure of the waveguide leads to frequency independent group delay, which enables an undistorted signal transmission at 25 Gbps. In addition, an advanced modulation scheme could be adapted due to the low-dispersion E-TUBE channel, which can increase the throughput of the link. A low-cost low-loss broadband dielectric waveguide solution referred to as an E-TUBE is proposed for replacing the existing electrical and optical interconnects in high throughput links, including but not limited to, 100/400 Gbps board-to-board communications.

## Methods

### S-parameter measurement

The S-parameter measurement is performed using an Agilent N5227A Network Analyzer with Agilent N5250CX10 110-GHz Frequency Extender Modules. The signal is launched into the DUT via 1.00-mm connectors (Southwest Microwave, Inc.). Both ports of the Agilent N5250CX10s are connected to the two ports of the DUT for 2-port S-parameter measurement. The transmission and reflection characteristics can be measured from 10 MHz to 110 GHz.

### Eye diagram and bit-error-rate measurement

The measurement setup for data transmission is shown in Supplementary Note 3. A transmitter IC is used for generating the RF signal by up-converting baseband signal (PRBS pattern) to the 70 GHz frequency band using a internally-generated carrier signal. The RF signal is transmitted to the channel which includes the Tx MWT, the E-TUBE, and the Rx MWT using 1.00-mm connectors and cables. The received RF signal through the channel is down-converted to the baseband using receiver IC in the opposite way.

## Supplementary information

Supplementary Information.
