# Supplementary material for: E-TUBE: dielectric waveguide cable for high-speed communication
Source: Sci Rep. 2020 Oct 26;10:18263. doi: 10.1038/s41598-020-75363-4 (PMC7589562; doi:10.1038/s41598-020-75363-4)
Supplement: Supplementary file 1 — Supplementary Information. [file 41598_2020_75363_MOESM1_ESM.pdf]

Supplementary Information for

E-TUBE: Dielectric waveguide cable for high-speed communication

Ha Il Song[1,2], Joon-Yeong Lee[2], Hyosup Won[2], Chang-Ahn Kim[2], Huxian Jin[2], Jake Eu[2], Jinho Park[2] and Hyeon-Min Bae[1*]

[1]Korea Advanced Institute of Science and Technology (KAIST), Department of Electrical Engineering, Daejeon, 34141, Republic of Korea

[2]Point2 technology Inc, Daejeon, 34051, Republic of Korea

*hmbae@kaist.ac.kr

Supplementary notes

1. Maximum transferable data rate

Channel capacity, the maximum amount of information that can be transmitted over a given channel, on the basis of Shannon's law is given by

$$C = \sum_{n=0}^{k} log_2\left(1 + \frac{IL(f)\times S(f)}{N_0}\right) \times \Delta f_n, \quad (1)$$

where f, S, IL and k denote the frequency, power spectrum, insertion loss of the channel and integration index of target frequency, respectively.

The practical channel capacity of the copper, the optical and E-TUBE links can be estimated by reflecting practical implementation issues and channel characteristics as well as plausible noise level reported by previous research. The power spectrum of the received signal is determined by the modulation scheme and the channel response. As a modulation scheme for high speed links, PAM (Pulse Amplitude Modulation) is a predominant method when considering implementation complexity and power efficiency. The Power Spectral Density (PSD) of PAM-N signaling is

$$\text{PAM2:} \quad S(f) = \frac{1}{4}A^2 T_b sinc^2(fT_b),$$

$$\text{PAM4:} \quad S(f) = \frac{5}{36}A^2 T_b sinc^2(fT_b),$$

$$\text{PAM8:}\quad S(f) = \frac{21}{392}A^2T_b sinc^2(fT_b),$$

$$\text{PAM16:}\quad S(f) = \frac{165}{14400}A^2T_b sinc^2(fT_b),$$

$$\text{PAM32:}\quad S(f) = \frac{286}{12008}A^2T_b sinc^2(fT_b),$$

$$\text{PAM64:}\quad S(f) = \frac{455}{1016064}A^2T_b sinc^2(fT_b),$$

$$\text{PAM128:}\quad S(f) = \frac{680}{8258048}A^2T_b sinc^2(fT_b),$$

where A denotes the signal amplitude, T_b is the baud duration and f is frequency.

The signal amplitude is limited by the power supply voltage, which is typically less than 2V. When the level of modulation is raised to increase the data rate without changing the baud rate, the amount of transmitted information saturates at some point since the signal-to-noise ratio (SNR) drops in inverse proportion to the modulation level. Therefore, there exists an optimum level of modulation in the PAM signaling scheme, which determines the maximum transferable data rate.

The copper links are grouped into two types: passive and active. Supplementary Figure 1 shows the description of such copper links.

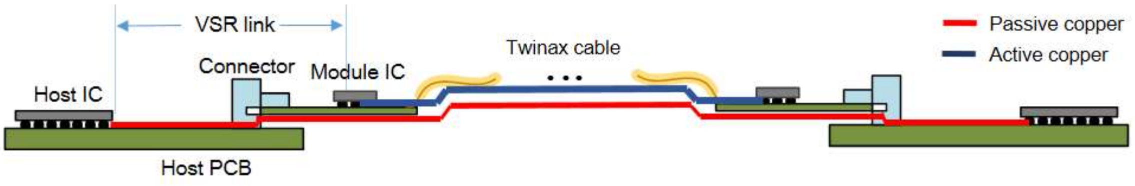

Supplementary figure 1 | Description of the copper channel. The blue and the red lines show the channel path of the active copper and the passive copper links respectively.

Typical passive copper links are composed of two very short reach (VSR) links and a Twinax cable, while the active copper link includes retimer ICs compensating for the dispersion of the copper channel and the Twinax cable. The channel paths of the passive copper and the active copper links are indicated by the blue and the red lines, respectively. The individual channel response of each link is shown on Supplementary Fig 2.

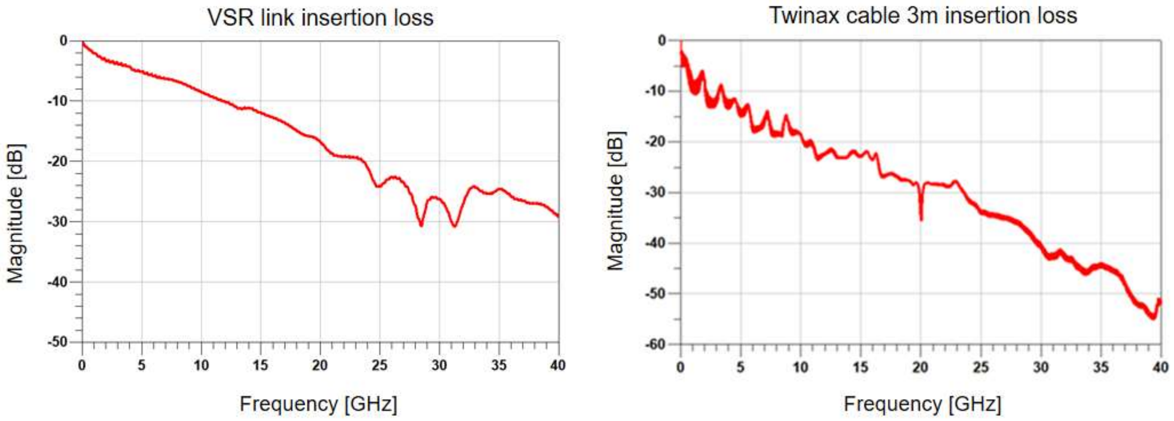

Supplementary figure 2 | Channel response of the copper link. Measured insertion loss of the Very Short Reach (VSR) link and the twinax cable[20].

The channel loss of a 3 m passive copper cable and a 3 m active copper cable is 96 dB and 46 dB, respectively at 28 GHz. The input-referred noise of a retimer IC can be inferred from input sensitivity of reported measurements [21-23]. The reported values are 17 mV_{ppd} at a data rate of 11.4 Gb/s[21], 42 mV_{ppd} at a data rate of 28 Gb/s[22] and 47 mV_{ppd} at a data rate of 56 Gb/s[23], which indicates the approximated noise floor of $0.24 \times 10^{-15}\ V^2/Hz$. From the measured channel response and estimated noise floor, the maximum transferable data rate of a 3 m active copper cable can be estimated.

In the PAM-N signaling, the data rate increases as the modulation index N increases. The maximum feasible N at a given symbol rate can be estimated by using Shannon's channel capacity. The data rate of PAM-N signal cannot exceed the channel capacity calculated from the PSD of the PAM-N signal in a given channel together with the estimated noise floor. For example, the data rate of PAM-32 signaling at a symbol rate of 14 GHz in a noiseless condition is 70 Gbps. However, the calculated channel capacity of the active copper channel is 38 Gbps when SNR condition is considered, which indicates that the PAM-32 signal at a baud rate of 14 GHz cannot be transmitted over 3 m active copper channel without errors. Likewise, the maximum data rate of PAM-N signaling can be estimated with respect to the symbol rate. Supplementary Fig.3 shows the highest data rate of PAM-N signal with respect to the baud rate.

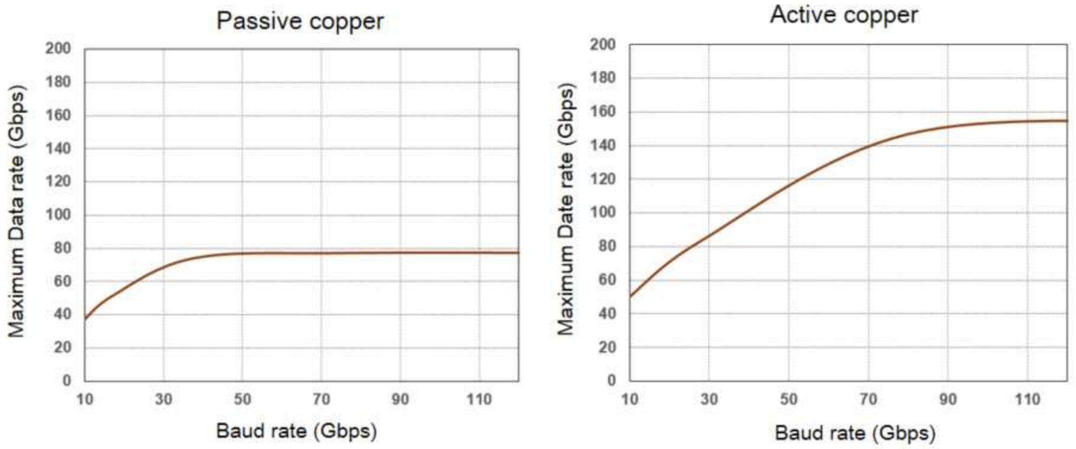

Supplementary figure 3 | The maximum data rate of the copper cable. The maximum data rate is determined by considering the loss and the noise characteristics of the channel link.

At low frequencies, the maximum data rate of the conductor-based interconnects grows in proportion to the baud rate. However, the maximum data rate saturates at high frequencies since no information can be transferred due to frequency-dependent channel loss. The estimated channel capacities of the passive and the active copper links are 78 Gbps and 156 Gbps, respectively

The optical link includes a retimer IC, optical transmit-and-receive components and an optical fiber. Typical optical-domain transmit power and SNR are 0 dBm and 20 dB, respectively and the bandwidth of an 3 m optical link is greater than 40 GHz with negligible loss and dispersion. The predominant noise sources in the optical link is the retimer IC as well as the transimpedance amplifier performing opto-electric conversion. The estimated maximum data rate of the optical link grows steadily with increasing baud rate (Supplementary Fig. 4).

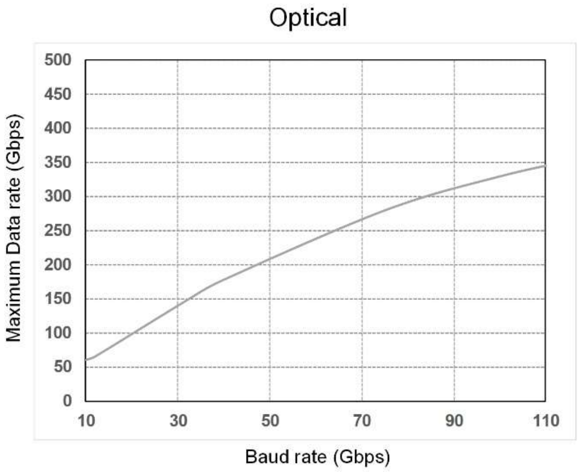

Supplementary figure 4 | The maximum data rate of the optical link. Estimated maximum data rate of an optical link when

PAM-N signaling is used.

The E-TUBE link includes a retimer IC, RF IC, the antenna and the E-tube. The E-tube itself has frequency independent loss profile in the passband. The bandwidth of a 3 m E-TUBE channel can be made greater than 40 GHz: Low 3-dB cutoff frequency is determined by geometry of the E-tube and the upper 3-dB cutoff frequency can be controlled via antenna design. The measured Tx output power is 10 dBm with the SNR of 35 dB and the Rx noise figure is 7 dB. The estimated maximum data rate of the E-TUBE link grows as the baud rate increases (Supplementary Fig. 5). Therefore, E-TUBE is a promising solution that can support rapidly growing demand for bandwidth of the high-speed short-reach communication links.

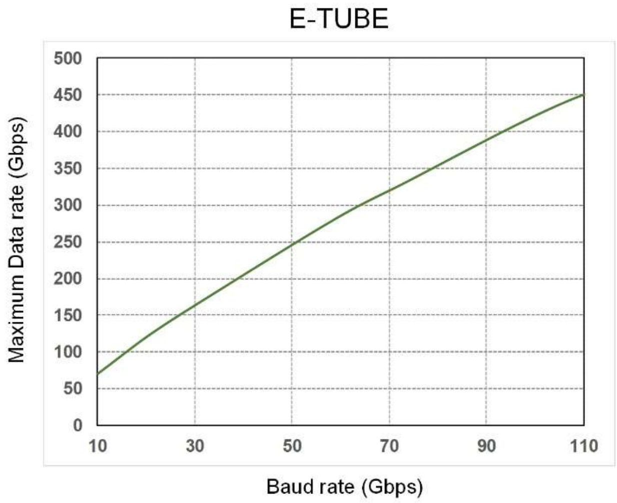

Supplementary figure 5 | The maximum data rate of the E-TUBE link. The maximum data rate is estimated by considering the loss and the noise characteristics of the channel link.

The actual maximum achievable data rate in using PAM-N signaling can vary when additional signal processing schemes including frequency/wavelength/polarization division multiplexing are employed.

2. Low Group velocity dispersion waveguide

Assume a plane wave with the property of frequency ω_0, wavenumber $k = nk_0$ and phase velocity $c = c_0/n$, where n is the refractive index of the dielectric core placed between two plates of the parallel plate waveguide is propagating as shown in Supplementary figure 6.

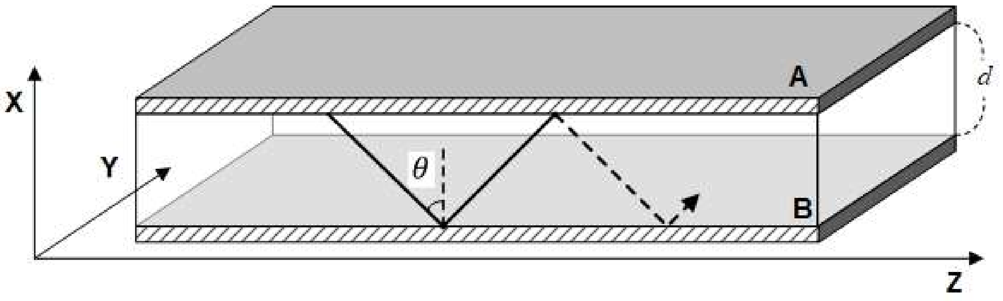

Supplementary Figure 6 | Parallel plate waveguide. A wave propagates through the waveguide consisting of two parallel plane conductors and the dielectric core.

The wave reflects back and forth between two metal boundaries while propagating in Z-direction. The condition for the wave propagation is determined by the superposition of the existing plane wave and the reflected-and-returned plane wave. In case constructive interference between the two waves is satisfied, the resultant wave maintains its appearance and propagate through the waveguide. Such condition is satisfied if the phase difference between two waves is identical or different by an integer multiple of 2π, as given by

$$k_x d + \pi + k_x d + \pi \ = 2\pi m_x \quad (m_x = 0, 1, 2\ ..). \tag{2}$$

where the separation between the two plates is d and k_x is the wavenumber in the direction of x.

This principle of the operation can be applied to a fully-enclosed metal waveguide with width and thickness of d_1 and d_2, respectively. The condition for the constructive interference in the fully-enclosed metal waveguide is

$$2k_x d_1 + 2\pi = 2\pi m_x \quad (m_x = 0, 1, 2..), \tag{3}$$

$$2k_y d_2 + 2\pi = 2\pi m_y \quad (m_y = 0, 1, 2..).$$

where k_x, k_y are the wavenumbers in the direction of x and y respectively.

In a single-mode waveguide, only one mode is allowed ($m_x = 0, m_y = 1$) and satisfies

$$k_x = \ -\frac{\pi}{d_1} \qquad k_y = \ 0. \tag{4}$$

The propagation constant of the guided wave is

$$\beta^2 = \ k^2 - k_x^2 - k_y^2 = \left({}^{\omega}/_{c_1}\right)^2 - \left({}^{\pi}/_{d_1}\right)^2. \tag{5}$$

According to the Maxwell equation, the rectangular waveguide satisfies $\omega^2 = \omega_c^2 + \beta^2 c^2$ where ω_c is cutoff frequency of the waveguide. By taking derivative of both sides, we get $2\omega d\omega = 2c^2 \beta d\beta$ and the group delay defined by $d\beta/d\omega$ is

$$\text{Group delay} = \frac{d\beta}{d\omega} = \frac{\omega}{c_1^2\beta} = \frac{\omega}{c_1^2}\frac{1}{\sqrt{\left(\frac{\omega}{c_1}\right)^2-\left(\frac{\pi}{d_1}\right)^2}} = \frac{1}{c_1\sqrt{1-\left(\frac{c_1\pi}{\omega d_1}\right)^2}} \propto \frac{{}^{1}/_{c_1}}{\sqrt{1-\frac{a}{\omega^2}}}. \quad (6)$$

The equation shows that the group delay of the fully-enclosed metal waveguide is inversely proportional to the frequency and the variation increases in the vicinities of the cutoff frequency.

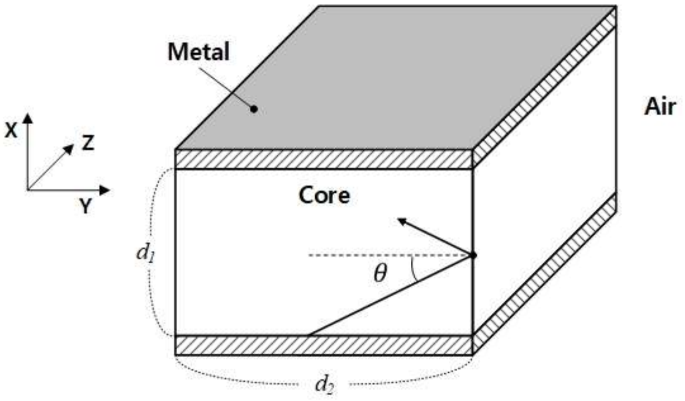

Supplementary Figure 7 | Partially-enclosed metal waveguide. A wave is propagating through the dielectric core placed between the two parallel plate waveguide.

On the other hands, the condition for the constructive interference in a proposed partially-enclosed metal waveguide is

$$2k_x d_1 + 2\pi = 2\pi m_x \quad (m_x = 0, 1, 2..), \quad (7)$$

$$2k_y d_2 + 2\varphi = 2\pi m_y \quad (m_y = 0, 1, 2..), \quad .$$

where the phase shift, φ, occurring at the boundary between the dielectric core and the air satisfies

$$\tan\left(\frac{\varphi}{2}\right) = \left(\frac{\sin^2\overline{\theta_c}}{\sin^2\theta} - 1\right)^{1/2}. \quad (8)$$

The angles θ and θ_c denote the incident angle from the core to the air and the corresponding critical angle, respectively. As shown in the supplementary figure 7, the incident angle θ can be identified by using k and k_x as

$$\sin\theta = \frac{\omega_c}{\omega}, \ \cos\theta = \sqrt{1 - \frac{\omega_c^2}{\omega^2}}. \quad (9)$$

For the partially-enclosed metal waveguide, single-mode wave propagation is ensured when $(m_x = 0, m_y = 1)$

$$k_x = -\frac{\varphi}{d_1} \qquad k_y = 0, \quad (10)$$

and the propagation constant of the guided wave is

$$\beta^2 = k^2 - k_x^2 - k_y^2 = \left({}^{\omega}/_{c_1}\right)^2 - \left({}^{\varphi}/_{d_1}\right)^2. \quad (11)$$

The group delay of the partially enclosed waveguide is

$$\text{Group delay} = \frac{d\beta}{d\omega} = \frac{\omega}{c_1^2\beta} = \frac{\omega}{c_1^2}\frac{1}{\sqrt{\left(\frac{\omega}{c_1}\right)^2-\left(\frac{\varphi}{d_1}\right)^2}} = \frac{1}{c_1\sqrt{1-\left(\frac{c_1\varphi}{\omega d_1}\right)^2}} \quad . \tag{12}$$

By substituting Supplementary equation 8 for Supplementary equation 12, the relation between the group delay and the frequency can be derived. Supplementary figure 8 shows the relation between the group delay and the frequency.

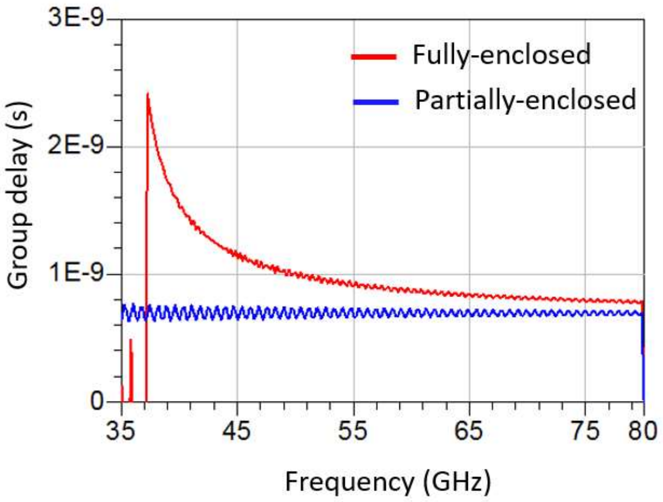

Supplementary figure 8 | Comparison of the group delay for the fully-enclosed waveguide and the partially-enclosed waveguide

It clearly shows that the group delay of the partially-enclosed metal waveguide remains constant over the passband as compared to that of the fully-enclosed metal waveguide.

3. Eye diagram and Bit-error-rate measurement

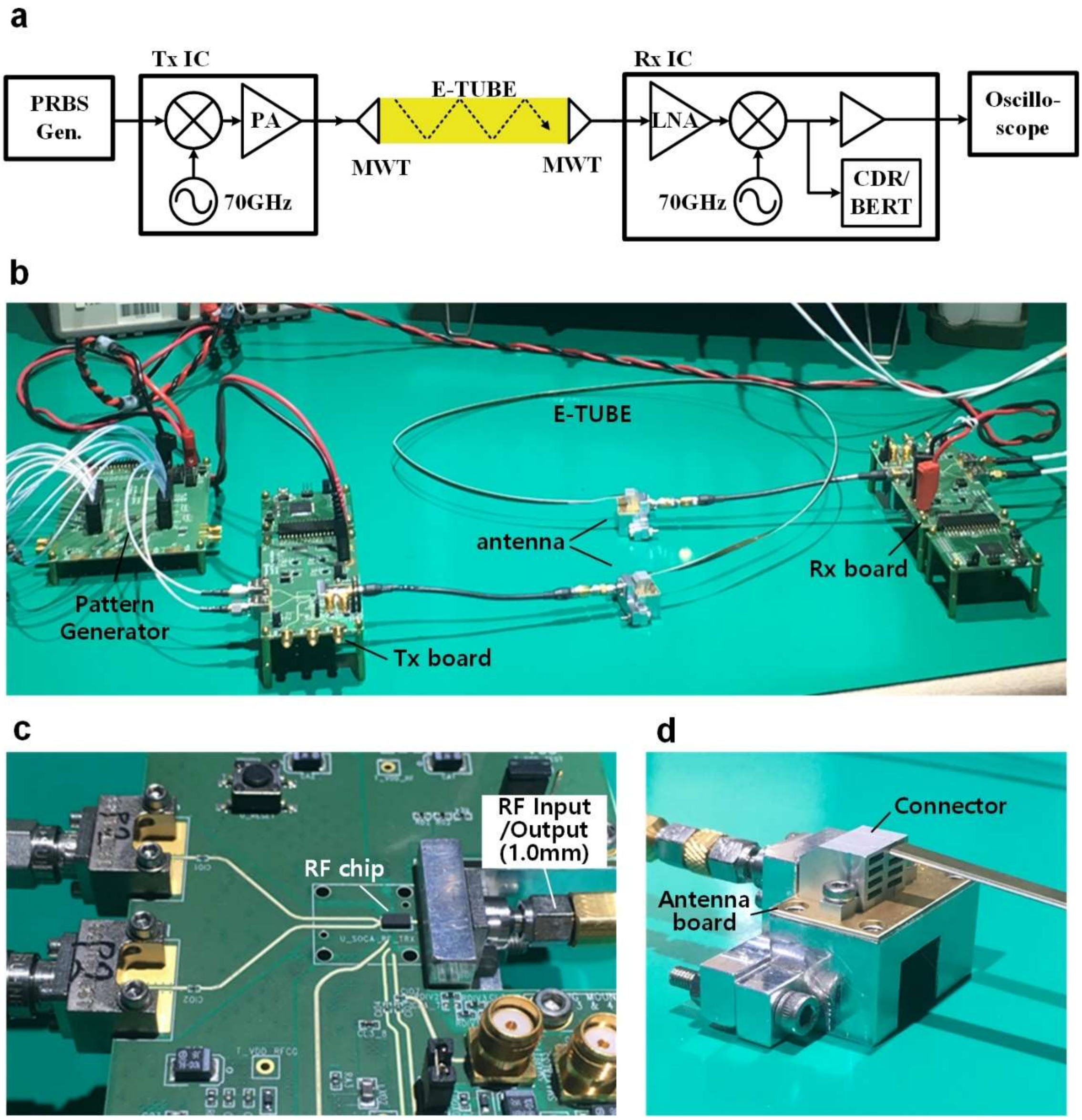

Supplementary figure 9 | (a) A schematic diagram of eye diagram and BER measurement setup. **(b)** Photographs of eye diagram measurement setup **(c)** RF board. 1.0mm connectors and the cables are utilized to connect the RF chips and the transition. **(d)** The etube-to-board connector is coupled to the board and the etube is inserted horizontally into the opening slot of the connector.

4. Bending performances

The bending performances of the E-TUBE can be compared with the existing interconnects by using the parameters such as bending radius and group delay variation.

The electrical domain bending performance of the existing metallic interconnects referred to as direct attached cable (DAC) is expected to be excellent as long as the cable maintains its designed geometry. As the covering data-rate increases, the stiffness of a DAC increases to due geometry, which eventually limits its bending

performance. The typical maximum bending radius of a 25GbE DAC is around 30mm. Precise electrical domain bending performance beyond the bending limit is not well defined since the degradation varies significantly depending on the type and degree of deformation of the cable.

The optical interconnects relies on the phenomenon of total internal reflection between the dielectric core and the dielectric cladding. If the bending degree increases, the propagating wave leaks out the core and thereby increasing the bending loss. Supplementary Figure 10 shows the measured bending loss of a single mode fiber [4-6]. In case of the standard single mode fiber, the minimum bending radius showing the loss of 1dB/turn is about 10mm. And the state-of-the-art paper reported that the nano-engineered fibers can reduce the bending radius down to below 5mm. In addition, the group velocity dispersion of the optical fiber can be negligible for a few meter data transmission even in the bended condition.

The E-TUBE suppresses the bending loss by using the reflection at the boundary between the metal cladding and the dielectric core. Supplementary Figure 11 shows the comparison between the straight E-TUBE and the bended E-TUBE, which shows that the loss from a 5mm single-turn bending is negligible. The measured group delay of a bended E-TUBE exhibits that the frequency-independency is maintained.

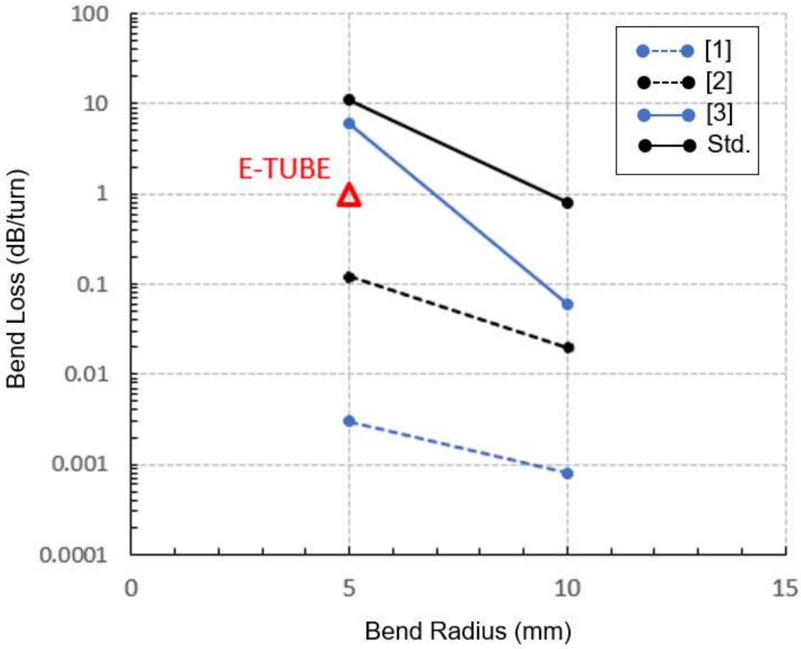

Supplementary Figure 10 | Comparison of bending performances of the optical interconnects and the E-TUBE.

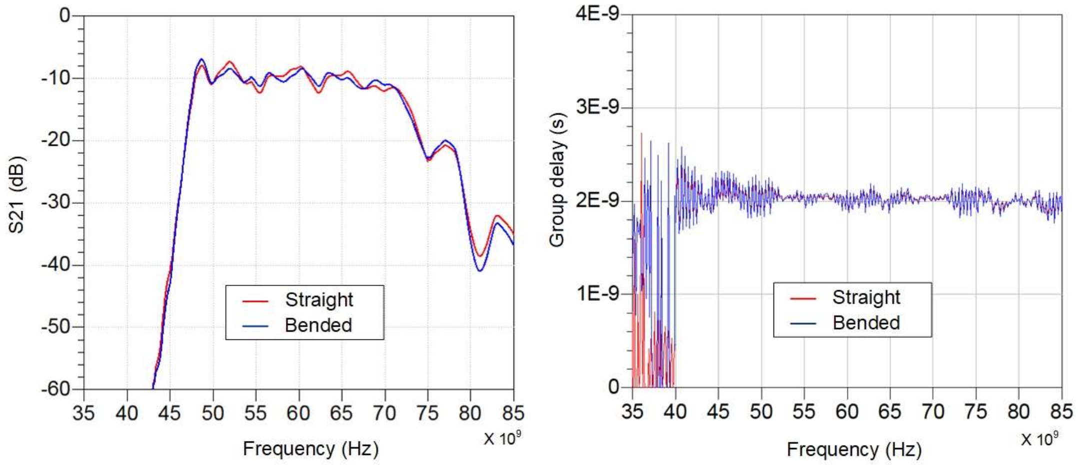

Supplementary Figure 11 | Bending performances of E-TUBE. Measured results for a 0.5 m bended E-TUBE whose bending radius is 5.0 mm and for a 0.5 m straight E-TUBE.

Consequently, the E-TUBE shows the comparable level of bending performances against the existing interconnects although there are a couple of uncertainties to compare the actual bending performances.

Supplementary references

1. IEEE 802.3 50Gb/s, 100Gb/s, and 200Gb/s Ethernet Task Force Contributed Channel Data, *http://grouper.ieee.org/groups/802/3/cd/public/channel/index.html*

2. A. Nazemi et al., A 2.8 mW/Gb/s quad-channel 8.5–11.4 Gb/s quasi-digital transceiver in 28 nm CMOS, 2013 Symposium on VLSI Circuits, Kyoto, 2013, pp. C276-C277.

3. H. Won et al., A 0.87 W Transceiver IC for 100 Gigabit Ethernet in 40 nm CMOS, in IEEE Journal of Solid-State Circuits, vol. 50, no. 2, pp. 399-413, Feb. 2015.

4. J. Lee et al., 56Gb/s PAM4 and NRZ SerDes transceivers in 40nm CMOS, 2015 Symposium on VLSI Circuits (VLSI Circuits), Kyoto, 2015, pp. C118-C119

5. Li, M-J., et al. "Ultra-low bending loss single-mode fiber for FTTH." Journal of Lightwave Technology 27.3 (2009): 376-382.

6. L.-A. de Montmorillon, P. Matthijsse, F. Gooijer, F. Achten, D. Molin, N. Montaigne, and J. Maury, "Bend-optimized G.652D compatible trench-assisted single-mode fibers," in Proc. 55th IWCS/Focus, Nov. 2006, pp. 342–347.

7. Himeno, Kuniharu, et al. "Low-bending-loss single-mode fibers for fiber-to-the-home." Journal of Lightwave Technology 23.11 (2005): 3494.
